# Comparative Evaluation of Microbial Adhesion and Surface Morphology of Bonded and Non‐Bonded Resin‐Based Composite Materials: A SEM and Microbiological Study

**DOI:** 10.1155/ijod/6327486

**Published:** 2026-06-10

**Authors:** Musab Hamed Saeed, Hafiz Ahmad, Lovely M. Annamma, Tareq Aljafarawi, Melahat Gorduysus, Sudhir Rama Varma, Mehmet Omer Gorduysus, Esraa Jaber

**Affiliations:** ^1^ Department of Clinical Sciences, College of Dentistry, Ajman University, Ajman, UAE, ajman.ac.ae; ^2^ Department of Microbiology, RAK College of Medical Sciences (RAKCOMS), RAK Medical and Health Sciences University (RAKMHSU), Ras Al Khaimah, UAE, rakmhsu.com; ^3^ Dental Research Cell, Dr. D. Y. Patil Dental College and Hospital, Dr. D. Y. Patil Vidyapeeth (Deemed to be University), Pune, 411018, Maharashtra, India, dpu.edu.in; ^4^ Department of Endodontics, Faculty of Dentistry, Cyprus Health and Sciences University, Morphou, Cyprus; ^5^ Department of Preventive and Restorative Dentistry, College of Dental Medicine, University of Sharjah, Sharjah, UAE, sharjah.ac.ae

**Keywords:** dental bonding agents, microbial adhesion, resin-based composites, scanning electron microscopy, surface morphology

## Abstract

**Objective:**

To evaluate the effect of applying bonding agents to uncured resin‐based composite surfaces on microbial adhesion and surface morphology.

**Materials and Methods:**

Five resin‐based composite materials (Clearfil AP‐X Esthetics, Prime Dent, Beautifil II, Saremco microhybrid composite [MHC], and Saremco extra‐low shrinkage composite [ELS]) were evaluated under bonded (test) and non‐bonded (control) conditions (*n* = 7 per group). In the bonded groups, a bonding agent was applied to the uncured composite surface prior to polymerization, whereas non‐bonded specimens were polymerized and subsequently polished. *Streptococcus mutans* adhesion was assessed using colony‐forming unit (CFU) analysis, and surface morphology was evaluated by scanning electron microscopy (SEM). Statistical analysis was performed using the Mann–Whitney *U* test (*α* = 0.05).

**Results:**

Bonded specimens exhibited significantly higher microbial adhesion than non‐bonded specimens for Saremco MHC, Saremco ELS, and Clearfil AP‐X Esthetics. In contrast, Prime‐Dent and Beautifil II showed lower CFU values in the bonded groups compared with the non‐bonded controls. Statistically significant differences between bonded and non‐bonded groups were observed for all tested composite materials (Mann–Whitney *U* test, *p*  < 0.01). SEM analysis revealed increased surface irregularities, micro‐retentive features, and heterogeneous topography on bonded surfaces, whereas non‐bonded specimens exhibited comparatively smoother and more homogeneous surface morphologies.

**Conclusion:**

Application of bonding agents to uncured composite resin surfaces alters surface morphology and is associated with altered microbial adhesion. These findings suggest that the clinical use of bonding agents as modeling liquids may influence the biological performance of composite restorations and should therefore be considered with caution.

## 1. Introduction

The global burden of untreated dental caries continues to be substantial, affecting ~2.3 billion individuals worldwide [1]. Resin‐based composite materials are widely employed in the restoration of carious lesions due to their favorable esthetic qualities and acceptable mechanical performance. However, their long‐term clinical success may be compromised by factors such as polymerization shrinkage, marginal discrepancies, and the development of secondary caries [2]. Among these, biofilm accumulation at restoration margins is considered a key contributor to the initiation and progression of recurrent caries [3]. The surface characteristics of restorative materials—particularly surface roughness and topography—are known to influence microbial adhesion, with smoother surfaces generally demonstrating reduced biofilm formation following polishing procedures [4].

Among oral microorganisms, *Streptococcus mutans* plays a central role in the cariogenic process due to its strong adhesion capability and acid‐producing potential [5–7]. These bacteria are capable of colonizing not only natural tooth structures but also the surfaces of restorative materials, thereby increasing the susceptibility to secondary caries at restoration interfaces [8–13].

Resin‐based composites consist of a polymerizable organic matrix, inorganic filler particles, and coupling agents that facilitate bonding between these components. Common monomers include bisphenol A‐glycidyl methacrylate (Bis‐GMA), triethylene glycol dimethacrylate (TEGDMA), urethane dimethacrylate (UDMA), and related compounds. Variations in filler size and distribution—ranging from macrofill to nanofill systems—can significantly influence the physical, mechanical, and biological behavior of these materials [14]. Certain monomers, particularly TEGDMA and ethylene glycol dimethacrylate (EGDMA), have been reported to enhance the growth of cariogenic microorganisms such as *Streptococcus sobrinus* and *Lactobacillus acidophilus* [15]. In addition, resin components have been extensively investigated for their potential cytotoxic and genotoxic effects [16–19]. Evidence from recent in vivo and animal studies further suggests that degradation and metabolic processes may lead to systemic exposure to monomers such as hydroxyethyl methacrylate (HEMA), TEGDMA, and Bis‐GMA following ingestion [20]. These concerns have driven the development of modified composite formulations, including low‐ and extra‐low‐shrinkage materials, aimed at reducing monomer release while maintaining functional properties.

Beyond intrinsic material composition, surface characteristics remain a critical determinant of bacterial colonization and biofilm development. Increased surface roughness and heterogeneity provide favorable conditions for microbial retention and maturation. Accordingly, finishing and polishing procedures are routinely recommended to minimize these surface irregularities. In clinical practice, however, bonding agents are frequently applied as modeling liquids to improve handling characteristics, reduce instrument adhesion, and facilitate incremental placement of composite resins [21–23].

While the use of bonding agents as modeling liquids may enhance clinical handling, their application to uncured composite surfaces has the potential to modify surface chemistry, polymerization behavior, and final surface morphology. Biofilm accumulation at restoration margins has been associated with factors such as polymerization stress, surface degradation, and breakdown of ester bonds within the resin matrix [24]. In particular, *Streptococcus mutans* has been shown to contribute to the degradation of resin‐based materials, leading to increased surface irregularity and enhanced bacterial colonization [25–27]. Despite existing research evaluating the effects of bonding or modeling agents on mechanical and optical properties, there remains a relative lack of data regarding their influence on microbial adhesion, especially when applied prior to polymerization. Moreover, few studies have directly compared bonded and non‐bonded composite surfaces across materials with different filler compositions in terms of both microbial behavior and surface morphology.

Therefore, the aim of the present study was to evaluate the effect of applying bonding agents to uncured composite resin surfaces on microbial adhesion and surface characteristics. Five commercially available composite materials, including low‐ and extra‐low‐shrinkage formulations, were investigated using *Streptococcus mutans* adhesion analysis and scanning electron microscopy (SEM). The null hypothesis was that the application of a bonding agent to uncured composite resin surfaces does not influence microbial adhesion or surface morphology.

## 2. Materials and Methods

Five commercially available resin‐based composite materials with different resin matrix formulations and filler compositions were evaluated in this study: Clearfil AP‐X Esthetics (Kuraray Noritake Dental Inc. 1621 Sakazu, Kurashiki, Okayama 710‐0801, Japan), Prime Dent (Prime‐Dent Composite, Prime Dental Manufacturing Inc., Chicago, IL, USA), Beautifil II (Shofu Inc., Kyoto, Japan), Saremco microhybrid composite resin (MHC) (SAREMCO Dental AG, Rebstein, Switzerland), and Saremco extra‐low shrinkage composite resin (ELS) (SAREMCO Dental AG, Rebstein, Switzerland). The composition, manufacturer, and filler characteristics of each composite material are summarized in Table 1. Bonding agents were selected according to the manufacturer’s recommendations. A Saremco ELS bonding agent was used for the Saremco ELS composite, while a single‐component bonding agent (James 2 Bond, Saremco Dental AG, Rebstein, Switzerland) was used for the remaining composite materials.

**Table 1:** Composite resins and bonding agents used in the study with their chemical composition and filler characteristics.

Material name	Type	Manufacturer	Composition	Particle size/filler content
CLEARFIL AP‐X Esthetics	Composite (microhybrid)	Kuraray Noritake Dental Inc., Japan	Monomers: BisGMA, TEGDMA, UDMA. Fillers: Silanated barium glass, silica, colloidal silica. Other: dl‐Camphorquinone, catalysts, accelerators, pigments	0.02–17 µm Filler: 86.5% w/w, 71% v/v
Prime‐Dent nano‐hybrid composite	Composite (nano‐hybrid)	Prime‐Dent Manufacturing, Inc., USA	Monomers: Bis‐GMA, Urethane dimethacrylate. Fillers: Ultrafine glass, glass/silica nanocluster	~0.7 µm Filler: 74% w/w
Saremco extra low shrinkage	Composite	Saremco Dental AG, Rebstein, Switzerland	Monomers: BisGMA, BisEMA. Fillers: Barium glass, silica. Other: Catalysts, inhibitors, pigments, free of TEGDMA and HEMA	0.04–3.00 µm Median: 0.7 µm Filler: 74% w/w, 49% v/v
Saremco microhybrid composite	Composite (microhybrid)	Saremco Dental AG, Rebstein, Switzerland	Monomers: Aromatic urethane methacrylate, BisEMAFillers: Barium glass, silica. Other: Silica catalyst, inhibitors, pigments	0.04–3.00 µm Median: 0.7 µm Filler: 74% w/w, 49% v/v
Beautifil II	Composite (nano‐hybrid)	Shofu Dental Corp., Kyoto, Japan	Resin‐based with S‐PRG (Surface pre‐reacted glass ionomer) fillers	0.01–4.0 µm average: 0.8 µm Filler: 83% w/w, 69% v/v
ELS unibond	Bonding agent	Saremco Dental AG, Rebstein, Switzerland	Ethanol, water, BisEMA, methacrylated phosphoric salt, initiators	Self‐etching adhesive (no filler)
James‐2 bond	Bonding agent	Saremco Dental AG, Rebstein, Switzerland	Hydroxyethyl methacrylate, urethane methacrylate, polyalkenoate methacrylized, hydroxypropyl methacrylate, glycerinedimethacrylate	Primer and bonding agent (no filler)

### 2.1. Specimen Preparation

A cylindrical silicone mold measuring 5 mm in diameter and 5 mm in height was used to fabricate composite resin specimens. For each composite material, 14 specimens were prepared and randomly assigned to two surface‐condition groups (*n* = 7 per group): bonded (test) and non‐bonded (control). The composite material was incrementally placed into the mold and carefully adapted to minimize air entrapment.

In the bonded groups, a thin layer of bonding agent was applied directly to the uncured composite resin surface using a microbrush, followed by light polymerization according to the manufacturer’s instructions. In the non‐bonded groups, specimens were polymerized without the application of a bonding agent.

Following polymerization, non‐bonded specimens were finished and polished using a standardized multi‐step polishing system (Sof‐Lex XT polishing discs; 3M ESPE, USA) in a sequential manner under constant pressure. Bonded specimens were intentionally left unpolished to preserve the surface characteristics produced by the bonding agent. Specimens exhibiting surface defects, voids, or visible irregularities were excluded from the study.

### 2.2. Experimental Design and Grouping

**Figure 1:**
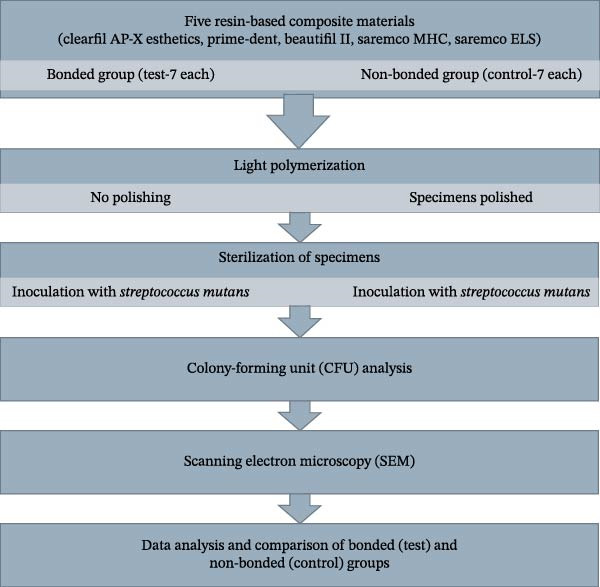
Flowchart illustrating the experimental design and workflow.

After surface preparation, specimens from both bonded and non‐bonded groups were stored under standardized conditions and subsequently subjected to microbial inoculation. This experimental design resulted in a total of 10 subgroups, comprising five composite materials within both the test and control groups (*n* = 7 per subgroup). A schematic representation of the experimental workflow and grouping is presented in Figure 1.

### 2.3. Microbiological Procedures

All specimens were sterilized by autoclaving prior to microbiological testing. Each specimen was individually placed in sterile 2 mL screw‐capped tubes containing 500 µL of artificial saliva (Moi‐stir) and incubated for 1 h to simulate oral conditions.

Subsequently, specimens were transferred to fresh tubes containing 1 mL of brain heart infusion (BHI) broth supplemented with 1% (w/v) sucrose. A 10 µL aliquot of an overnight culture of *Streptococcus mutans* (ATCC 25175) was added to each tube. The specimens were then incubated at 37°C for 72 h to allow biofilm formation.

Following incubation, the culture medium was carefully aspirated, and the specimens were rinsed three times with sterile distilled water to remove non‐adherent bacterial cells. Each specimen was then transferred to a tube containing 1 mL of sterile saline and vortexed for 3 min to detach adherent bacteria.

### 2.4. Quantification of Microbial Adhesion

The resulting bacterial suspension from each specimen was serially diluted (1:10), and microbial adhesion was quantified using the Miles and Misra technique. TYCSB (Tryptone Yeast Extract Cystine Sucrose Bacitracin) agar plates were divided into labeled sectors corresponding to the dilution factors. A 20 µL aliquot from each dilution was dispensed onto the agar surface and allowed to spread naturally.

The plates were then air‐dried, inverted, and incubated in a candle jar at 37°C for 18–24 h. Colonies were counted in sectors containing discrete and countable colonies (2–20 colonies per sector). Colony‐forming units per milliliter (CFU/mL) were calculated using the following formula:

CFU/mL= Average colony count×50× dilution factor.

### 2.5. Scanning Electron Microscopy (SEM)

Following microbiological analysis, one representative specimen from each experimental subgroup was selected for surface morphological evaluation. The specimens were dehydrated, sputter‐coated with gold, and examined using SEM to assess surface topography, irregularities, and micro‐retentive features.

**Figure 2:**
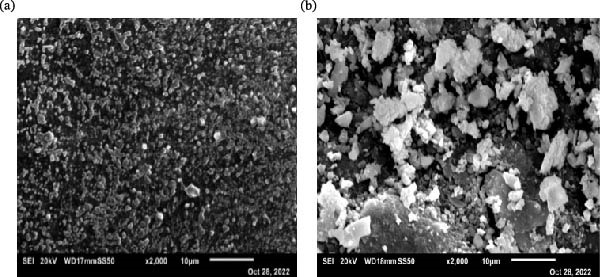
Beautifil II: (a) Surface morphology in bonded Beautifil II (Test). (b) Surface morphology in non‐bonded Beautifil II (control).

**Figure 3:**
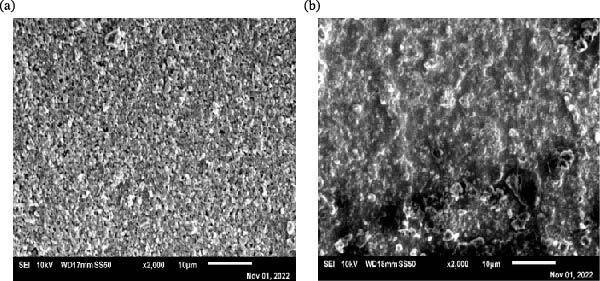
Saremco MHC: (a) Surface morphology in bonded Saremco MHC (Test). (b) Surface morphology in non‐bonded Saremco MHC (control).

**Figure 4:**
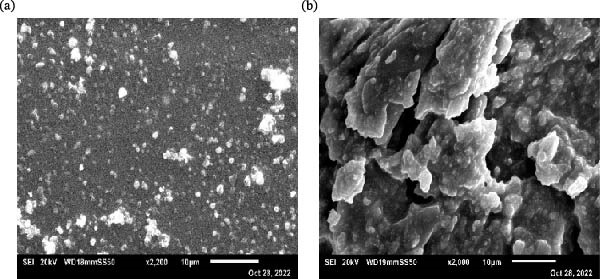
Clearfil: (a) Surface morphology in bonded Clearfil AP‐X Esthetics (Test). (b) Surface morphology in non‐bonded Clearfil AP‐X Esthetics (control).

**Figure 5:**
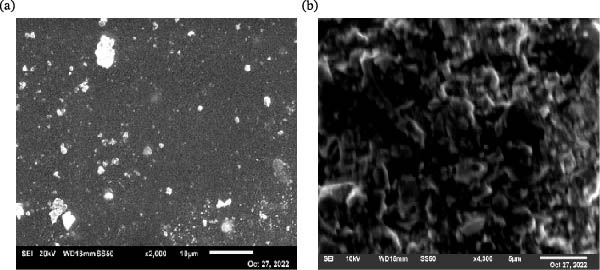
Prime‐Dent: (a) Surface morphology in bonded Prime‐Dent (Test). (b) Surface morphology in non‐bonded Prime‐Dent (control).

**Figure 6:**
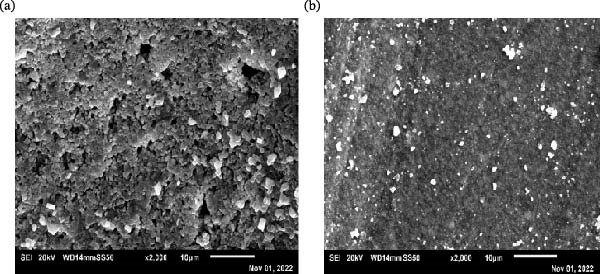
Saremco ELS: (a) Surface morphology in bonded Saremco ELS (Test). (b) Surface morphology in non‐bonded Saremco ELS (control).

SEM images were used for qualitative assessment of surface morphology and biofilm distribution. No quantitative scoring system was applied; therefore, interobserver agreement analysis was not required. Representative SEM micrographs are presented in bonded specimens (Figures 2a–6a) exhibited heterogeneous and irregular surface topography, whereas non‐bonded specimens (Figures 2b–6b) demonstrated smoother and more homogeneous surfaces.

### 2.6. Statistical Analysis

Data normality was evaluated using the Shapiro–Wilk test, which indicated a non‐normal distribution (*p*  < 0.01). Accordingly, comparisons between bonded and non‐bonded groups were performed using the Mann–Whitney *U* test. The level of statistical significance was set at *α* = 0.05.

## 3. Results

### 3.1. Microbiological Results

Microbial colony counts (CFU/mL) differed between bonded (test) and non‐bonded (control) groups across all evaluated composite materials. Data were non‐normally distributed; therefore, results are presented as median and interquartile range (IQR) and analyzed using the Mann–Whitney *U* test. Median (IQR) values and statistical comparisons are summarized in Table 2.

**Table 2:** Statistical comparison of microbial adhesion between bonded and non‐bonded groups (Mann–Whitney *U* test).

Composite	Bonded median (IQR)	Control median (IQR)	*U* value	*p*‐Value
Saremco MHC	189,090 (174,950–220,020)	86,660 (86,305–86,665)	49	0.002
Saremco ELS	215,000 (163,150–243,260)	92,120 (92,110–92,135)	49	0.002
Clearfil	181,000 (125,965–200,895)	101,600 (101,200–101,610)	49	0.002
Prime‐dent	25,280 (21,308–26,080)	96,050 (95,975–96,100)	0	0.002
Beautifil II	17,900 (14,720–19,370)	97,400 (96,905–97,405)	0	0.002

For Saremco MHC, the bonded group demonstrated a median CFU value of 189,090 (IQR: 174,950–220,020), compared with 86,660 (IQR: 86,305–86,665) in the non‐bonded group. Similarly, Saremco ELS showed a bonded median of 215,000 (IQR: 163,150–243,260) vs. 92,120 (IQR: 92,110–92,135) in the control group. Clearfil AP‐X Esthetics also exhibited higher microbial counts in the bonded group, with a median of 181,000 (IQR: 125,965–200,895), compared with 101,600 (IQR: 101,200–101,610) in the non‐bonded group.

In contrast, Prime‐Dent demonstrated lower microbial adhesion in the bonded group, with a median CFU value of 25,280 (IQR: 21,308–26,080), compared with 96,050 (IQR: 95,975–96,100) in the non‐bonded group. Similarly, Beautifil II showed reduced CFU values in the bonded group [17,900 (IQR: 14,720–19,370)] relative to the control group [97,400 (IQR: 96,905–97,405)].

Statistical analysis using the Mann–Whitney *U* test revealed significant differences between bonded and non‐bonded groups for all composite materials (*p*  < 0.01). For Saremco MHC, Saremco ELS, and Clearfil, higher microbial adhesion was observed in bonded specimens, whereas Prime‐Dent and Beautifil demonstrated the opposite trend, with lower adhesion in bonded groups.

These findings indicate that the effect of bonding agent application on microbial adhesion is material‐dependent, with consistent statistically significant differences across all tested composites.

### 3.2. SEM Analysis Results

SEM revealed distinct differences in surface morphology between bonded and non‐bonded composite resin specimens following biofilm exposure. Bonded specimens exhibited heterogeneous surface topography characterized by irregular surface patterns, exposed filler particles, and micro‐retentive features. In contrast, non‐bonded specimens demonstrated smoother and more homogeneous surface morphology with fewer irregularities and reduced biofilm accumulation.

SEM observations were qualitative in nature and served to support the microbiological findings. No quantitative surface roughness measurements were performed; therefore, these observations should be interpreted as descriptive rather than quantitative.

## 4. Discussion

The rationale for this study was based on the widespread clinical practice in which dentists and dental students use bonding agents as modeling liquids to facilitate manipulation of composite resins and reduce instrument adhesion. This reduction in stickiness is attributed to decreased surface tension, allowing improved incremental placement and contouring of the material while potentially minimizing void formation within the restoration [21, 28]. The most common difficulty related to composite resin sticking to instruments occurs during incremental layering [25]. Previous studies have demonstrated that the application of modeling or bonding agents can alter the surface and mechanical properties of resin‐based composites. Nantawan et al. reported reduced Vickers hardness values following bonding agent application after thermocycling [23], while Kutuk et al. showed that modeling agents can significantly influence surface roughness, microhardness, and color stability [29]. Collectively, these findings indicate that modeling agents may modify the surface characteristics of composite resins. The present study expands on this body of work by evaluating microbial adhesion rather than purely physical or optical properties, using newer composite formulations with modified resin matrices.

Earlier studies by Dunn et al. [30] reported negligible effects of isopropyl alcohol and unfilled resin on flexural strength when used as modeling media. However, subsequent investigations have demonstrated that bonding or modeling agents applied during composite placement can influence surface integrity and mechanical behavior [31, 32]. Bond strength, surface hardness, and interfacial stability have all been reported to be affected by such applications [33, 34]. While some studies suggest improved physical stability with reduced void formation [34], others report potential adverse effects related to surface modification [33]. These discrepancies highlight that the influence of modeling agents may depend on both material composition and application protocol. Additionally, concerns regarding the cytotoxic and genotoxic effects of resin monomers such as UDMA and TEGDMA remain relevant [35–37].

Most previous investigations have focused on physical and optical properties, including hardness, color stability, and water sorption [29, 38–40]. Modifications in resin composition, such as reduced Bis‐GMA content or substitution with Bis‐EMA, have been introduced to improve material performance. While these changes may influence mechanical behavior, their impact on microbial adhesion remains less well characterized. In this context, the present study provides additional insight by demonstrating that bonding agent application can significantly influence bacterial adhesion patterns.

The results of this study demonstrated that microbial adhesion differed significantly between bonded and non‐bonded surfaces for all tested composite materials (*p* ≈ 0.002). However, the direction of this effect was material‐dependent. Bonded specimens of Saremco MHC, Saremco ELS, and Clearfil exhibited higher microbial adhesion compared with their non‐bonded counterparts, whereas Prime‐Dent and Beautifil II showed reduced adhesion in the bonded groups. This consistent statistical significance across all materials, combined with variation in direction, suggests that the interaction between bonding agents and composite surface characteristics is complex and influenced by material composition.

*Streptococcus mutans* was selected due to its well‐established role in biofilm formation and secondary caries development. While previous studies have primarily evaluated physical surface properties, limited data exist regarding microbial adhesion to composite surfaces treated with bonding agents prior to polymerization. The present findings therefore contribute to filling this gap in the literature and do not support the null hypothesis.

The observed differences in microbial adhesion may be related to variations in surface topography and material composition. SEM analysis demonstrated that bonded specimens generally exhibited more heterogeneous and irregular surface morphologies compared with smoother, more uniform surfaces in non‐bonded specimens. These surface irregularities may increase the availability of micro‐retentive sites, thereby facilitating bacterial adhesion. However, the fact that some materials (e.g., Prime‐Dent and Beautifil II) demonstrated reduced adhesion despite bonding agent application suggests that additional factors, such as filler type, resin matrix composition, and surface chemistry, may also play a role.

Differences in microbial adhesion between materials may also reflect variations in filler distribution and surface microstructure. Previous studies have suggested that increased surface heterogeneity and microstructural irregularities can enhance bacterial retention [41, 42]. In the present study, materials exhibiting more pronounced surface irregularities appeared to demonstrate greater microbial adhesion; however, this relationship was not uniform across all composites, further supporting a material‐dependent effect.

Surface roughness is widely recognized as a key factor influencing bacterial adhesion. In this study, bonded specimens exhibited more irregular and heterogeneous surface features, including localized protrusions and uneven topography, which may contribute to increased bacterial retention. Nevertheless, as quantitative surface roughness measurements were not performed, these observations remain qualitative and should be interpreted with caution.

## 5. Limitations of the Study

Several limitations should be considered when interpreting these findings. First, differences in surface finishing between bonded (unpolished) and non‐bonded (polished) specimens introduce a confounding variable that may influence microbial adhesion independently of bonding agent application. Second, the relatively small sample size, although consistent with similar in vitro studies, may limit the generalizability of the results. Third, SEM analysis was qualitative in nature and did not include quantitative surface roughness measurements, which would have strengthened the correlation between surface topography and microbial adhesion. Additionally, the use of a single‐species biofilm model limits clinical extrapolation, as oral biofilms are inherently multispecies in nature. Future studies incorporating larger sample sizes, quantitative surface analysis, and multispecies biofilm models are recommended.

## 6. Conclusion

Within the limitations of this in vitro study, the application of bonding agents to uncured composite resin surfaces was associated with significant differences in microbial adhesion and alterations in surface morphology compared with non‐bonded surfaces. The effect of bonding agent application was material‐dependent, with both increases and decreases in microbial adhesion observed depending on the composite tested.

Possible mechanisms underlying these observations include the formation of surface irregularities, micro‐porosities, and interfacial changes that may influence bacterial retention. However, these mechanisms were not directly evaluated and should be interpreted cautiously.

Overall, the use of bonding agents as modeling liquids may influence the surface characteristics and microbial behavior of composite restorations under laboratory conditions. The clinical relevance of these findings remains uncertain, and further studies under clinically representative conditions are required.

## Funding

This study did not receive any specific funding.

## Disclosure

Esraa Jaber (corresponding author and manuscript guarantor) had full access to all of the data in this study and takes complete responsibility for the integrity of the data and the accuracy of the data analysis. All authors have read and approved the final version of the manuscript.

## Conflicts of Interest

The authors declare no conflicts of interest.

## Data Availability

The data that support the findings of this study are available from the corresponding author upon reasonable request.

## References

[1] Elamin A. and Ansah J. P. , Projecting the Burden of Dental Caries and Periodontal Diseases Among the Adult Population in the United Kingdom Using a Multi-State Population Model, Frontiers in Public Health. (2023) 11, 10.3389/fpubh.2023.1190197, 37744497. PMC10513470 37744497

[2] Aminoroaya A. , Esmaeely Neisiany R. , Nouri Khorasani S. , Panahi P. , Das O. , and Ramakrishna S. , A Review of Dental Composites: Methods of Characterizations, ACS Biomaterials Science & Engineering. (2020) 6, no. 7, 3713–3744, 10.1021/acsbiomaterials.0c00051. 33463319

[3] Mo S.-S. , Bao W. , Lai G.-Y. , Wang J. , and Li M.-Y. , The Microfloral Analysis of Secondary Caries Biofilm Around Class I and Class II Composite and Amalgam Fillings, BMC Infectious Diseases. (2010) 10, no. 1, 10.1186/1471-2334-10-241. PMC2931511 20712908

[4] Opdam N. J. , van de Sande F. H. , and Bronkhorst E. , et al.Longevity of Posterior Composite Restorations: A Systematic Review and Meta-Analysis, Journal of Dental Research. (2014) 93, no. 10, 943–949, 10.1177/0022034514544217. 25048250 PMC4293707

[5] de Soet J. J. , Nyvad B. , and Kilian M. , Strain–Related Acid Production by Oral Streptococci, Caries Research. (2000) 34, no. 6, 486–490, 10.1159/000016628. 11093023

[6] Marsh P. D. , Dental Plaque as a Biofilm and a Microbial Community—Implications for Health and Disease, BMC Oral Health. (2006) 6, no. S1, 10.1186/1472-6831-6-S1-S14. PMC2147593 16934115

[7] Hamada S. and Slade H. D. , Biology, Immunology, and Cariogenicity of *Streptococcus mutans* , Microbiological Reviews. (1980) 44, no. 2, 331–384, 10.1128/mr.44.2.331-384.1980. 6446023 PMC373181

[8] Sbordone L. and Bortolaia C. , Oral Microbial Biofilms and Plaque-Related Diseases: Microbial Communities and Their Role in the Shift From Oral Health to Disease, Clinical Oral Investigations. (2003) 7, no. 4, 181–188, 10.1007/s00784-003-0236-1. 14598129

[9] Svanberg M. , Mjör I. A. , and Ørstavik D. , Mutans Streptococci in Plaque From Margins of Amalgam, Composite, and Glass-Ionomer Restorations, Journal of Dental Research. (1990) 69, no. 3, 861–864, 10.1177/00220345900690030601. 2109000

[10] Krämer N. , Kunzelmann K-H. , García-Godoy F. , Häberlein I. , Meier B. , and Frankenberger R. , Determination of Caries Risk at Resin Composite Margins, American Journal of Dentistry. (2007) 20, no. 1, 59–64. 17380810

[11] Montanaro L. , Campoccia D. , and Rizzi S. , et al.Evaluation of Bacterial Adhesion of *Streptococcus mutans* on Dental Restorative Materials, Biomaterials. (2004) 25, no. 18, 4457–4463, 10.1016/j.biomaterials.2003.11.031. 15046936

[12] De Fúcio S. B. P. , Puppin-Rontani R. M. , De Carvalho F. G. , De Mattos-Graner R. O. , Correr-Sobrinho L. , and Garcia-Godoy F. , Analyses of Biofilms Accumulated on Dental Restorative Materials, American Journal of Dentistry. (2009) 22, no. 3, 131–136. 19650591

[13] Manhart J. , Chen H. , Hamm G. , and Hickel R. , Review of the Clinical Survival of Direct and Indirect Restorations in Posterior Teeth of the Permanent Dentition, Operative Dentistry. (2004) 29, no. 5, 481–508. 15470871

[14] Ferracane J. L. , Resin Composite—State of the Art, Dental Materials. (2011) 27, no. 1, 29–38, 10.1016/j.dental.2010.10.020. 21093034

[15] Hansel C. , Leyhausen G. , Mai U. E. H. , and Geurtsen W. , Effects of Various Resin Composite (Co)Monomers and Extracts on Two Caries-Associated Micro-Organisms In Vitro, Journal of Dental Research. (1998) 77, no. 1, 60–67, 10.1177/00220345980770010601. 9437400

[16] Huang F. M. , Chang Y. C. , and Lee S. S. , et al.BisGMA-Induced Cytotoxicity and Genotoxicity in Macrophages are Attenuated by Wogonin Via Reduction of Intrinsic Caspase Pathway Activation, Environmental Toxicology. (2016) 31, no. 2, 176–184, 10.1002/tox.22032. 26756871

[17] Durner J. , Schrickel K. , Watts D. C. , Becker M. , and Draenert M. E. , Direct and Indirect Eluates From Bulk Fill Resin-Based Composites, Dental Materials. (2022) 38, no. 3, 489–507, 10.1016/j.dental.2022.02.001. 35165002

[18] De Nys S. , Putzeys E. , and Duca R. C. , et al.Long-Term Elution of Bisphenol A From Dental Composites, Dental Materials. (2021) 37, no. 10, 1561–1568, 10.1016/j.dental.2021.08.005. 34482962

[19] Marzouk T. , Sathyanarayana S. , Kim A. S. , Seminario A. L. , and McKinney C. M. , A Systematic Review of Exposure to Bisphenol A From Dental Treatment, JDR Clinical & Translational Research. (2019) 4, no. 2, 106–115, 10.1177/2380084418816079. 30931707 PMC6728453

[20] Bakopoulou A. , Papadopoulos T. , and Garefis P. , Molecular Toxicology of Substances Released From Resin-Based Dental Restorative Materials, International Journal of Molecular Sciences. (2009) 10, no. 9, 3861–3899, 10.3390/ijms10093861. 19865523 PMC2769064

[21] Perdigão J. and Gomes G. , Effect of Instrument Lubricant on the Cohesive Strength of a Hybrid Resin Composite, Quintessence International. (2006) 37, no. 8, 621–625. 16922021

[22] Kosewski J. , Kosewski P. , and Mielczarek A. , Influence of Instrument Lubrication on Properties of Dental Composites, European Journal of Dentistry. (2022) 16, no. 4, 719–728, 10.1055/s-0042-1743144. 35395691 PMC9683889

[23] Krajangta N. , Ninbanjong S. , Khosook S. , Chaitontuak K. , and Klaisiri A. , Effects of Immediate Coating on Unset Composite With Different Bonding Agents to Surface Hardness, European Journal of Dentistry. (2022) 16, no. 4, 828–832, 10.1055/s-0041-1740221. 35181872 PMC9683890

[24] Mushashe A. M. , de Almeida S. A. , Ferracane J. L. , Merritt J. , and Correr G. M. , Effect of Biofilm Exposure on Marginal Integrity of Composite Restorations, American Journal of Dentistry. (2020) 33, no. 4, 201–205. 32794395 PMC8136684

[25] Nedeljkovic I. , De Munck J. , and Ungureanu A.-A. , et al.Biofilm-Induced Changes to the Composite Surface, Journal of Dentistry. (2017) 63, 36–43, 10.1016/j.jdent.2017.05.015. 28554609

[26] Huang B. , Cvitkovitch D. G. , Santerre J. P. , and Finer Y. , Biodegradation of Resin-Dentin Interfaces is Dependent on the Restorative Material, Mode of Adhesion, Esterase or MMP Inhibition, Dental Materials. (2018) 34, no. 9, 1253–1262, 10.1016/j.dental.2018.05.008. 29789163

[27] Steinberg D. and Eyal S. , Early Formation of *Streptococcus sobrinus* Biofilm on Various Dental Restorative Materials, Journal of Dentistry. (2002) 30, no. 1, 47–51, 10.1016/S0300-5712(01)00058-6. 11741735

[28] Li X. , Liu W. , and Sun L. , et al.Resin Composites Reinforced by Nanoscaled Fibers or Tubes for Dental Regeneration, BioMed Research International. (2014) 2014, 542958. 24982894 10.1155/2014/542958 PMC4058202

[29] Kutuk Z. B. , Erden E. , Aksahin D. L. , Durak Z. E. , and Dulda A. C. , Influence of Modeling Agents on the Surface Properties of an Esthetic Nano-Hybrid Composite, Restorative Dentistry & Endodontics. (2020) 45, no. 2, 10.5395/rde.2020.45.e13. PMC7239675 32483531

[30] Dunn W. J. and Strong T. C. , Effect of Alcohol and Unfilled Resin in the Incremental Buildup of Resin Composite, Quintessence International. (2007) 38, no. 1, e20–e26. 17508071

[31] Paolone G. , Direct Composites in Anteriors: A Matter of Substrate, The International Journal of Esthetic Dentistry. (2017) 12, no. 4, 468–481. 28983532

[32] Perdigão J. , Araujo E. , Ramos R. Q. , Gomes G. , and Pizzolotto L. , Adhesive Dentistry: Current Concepts and Clinical Considerations, Journal of Esthetic and Restorative Dentistry. (2021) 33, no. 1, 51–68, 10.1111/jerd.12692. 33264490

[33] Tuncer S. , Demirci M. , Tiryaki M. , Ünlü N. , and Uysal Ö. , The Effect of a Modeling Resin and Thermocycling on the Surface Hardness, Roughness, and Color of Different Resin Composites, Journal of Esthetic and Restorative Dentistry. (2013) 25, no. 6, 404–419, 10.1111/jerd.12063. 24172016

[34] Münchow E. A. , Sedrez-Porto J. A. , Piva E. , Pereira-Cenci T. , and Cenci M. S. , Use of Dental Adhesives as Modeler Liquid of Resin Composites, Dental Materials. (2016) 32, no. 4, 570–577, 10.1016/j.dental.2016.01.002. 26850844

[35] Carrillo-Cotto R. , Etges A. , and Jardim P. S. , et al.Cytotoxicity of Contemporary Resin-Based Dental Materials in Contact With Dentin, European Journal of Oral Sciences. (2020) 128, no. 5, 436–443, 10.1111/eos.12723. 32741041

[36] Schwengberg S. , Bohlen H. , and Kleinsasser N. , et al.In Vitro Embryotoxicity Assessment With Dental Restorative Materials, Journal of Dentistry. (2005) 33, no. 1, 49–55, 10.1016/j.jdent.2004.08.001. 15652168

[37] Geurtsen W. , Lehmann F. , Spahl W. , and Leyhausen G. , Cytotoxicity of Dental Resin Composite Monomers/Additives, Journal of Biomedical Materials Research. (1998) 41, no. 3, 474–480. 9659618 10.1002/(sici)1097-4636(19980905)41:3<474::aid-jbm18>3.0.co;2-i

[38] Sneed W. D. and Draughn R. A. , Effect of Alcohol on the Strength of a Composite Resin, Operative Dentistry. (1980) 5, no. 2, 47–48. 6937875

[39] Kleinsasser N. H. , Schmid K. , and Sassen A. W. , et al.Cytotoxic and Genotoxic Effects of Resin Monomers, Biomaterials. (2006) 27, no. 9, 1762–1770, 10.1016/j.biomaterials.2005.09.023. 16242184

[40] Schweikl H. , Spagnuolo G. , and Schmalz G. , Genetic and Cellular Toxicology of Dental Resin Monomers, Journal of Dental Research. (2006) 85, no. 10, 870–877, 10.1177/154405910608501001. 16998124

[41] Bollenl C. M. L. , Lambrechts P. , and Quirynen M. , Comparison of Surface Roughness of Oral Hard Materials to the Threshold for Bacterial Plaque Retention, Dental Materials. (1997) 13, no. 4, 258–269, 10.1016/S0109-5641(97)80038-3. 11696906

[42] Ionescu A. , Brambilla E. , and Wastl D. S. , et al.Influence of Matrix and Filler Fraction on Biofilm Formation on Resin-Based Composites, Journal of Materials Science: Materials in Medicine. (2015) 26, no. 1, 10.1007/s10856-014-5372-4, 5372. 25604698

